# Comparison of Radionuclide Impurities Activated during Irradiation of ^18^O-Enriched Water in Tantalum and Silver Targets during the Production of ^18^F in a Cyclotron

**DOI:** 10.3390/molecules28083485

**Published:** 2023-04-14

**Authors:** Teresa Jakubowska, Magdalena Długosz-Lisiecka, Michał Biegała

**Affiliations:** 1 Institute of Applied Radiation Chemistry, Faculty of Chemistry, Lodz University of Technology, Wróblewskiego 15, 90-924 Łódź, Poland; 2 Department of Medical Physics, Copernicus Memorial Hospital in Lodz Comprehensive Cancer Center and Traumatology, Pabianicka 62, 93-513 Łódź, Poland; 3 Department of Medical Imaging Technology, Medical University of Lodz, ul. Lindleya 6, 690-131 Łódź, Poland

**Keywords:** tantalum target body, silver target body, cyclotron, gamma spectrometry, activation product, FDG

## Abstract

During the production of ^18^F, as a result of the interaction of the beam of protons and secondary neutrons with the structural elements of the target body, many radionuclide impurities are created in the cyclotron. As part of this work, we theoretically predicted which isotopes would be activated in the target tantalum or silver bodies. Subsequently, we used gamma spectrometry analysis to verify these predictions. The results were compared with the work of other authors who studied titanium and niobium as materials for making the target body. Tantalum has been evaluated as the most favorable in terms of generating radionuclide impurities during the production of ^18^F by irradiation of ^18^O-enriched water in accelerated proton cyclotrons. Only three radionuclides were identified in the tested samples: ^181^W, ^181^Hf, and ^182^Ta with a half-life of fewer than 120 days. The remaining reactions led to the formation of stable isotopes.

## 1. Introduction

The ^18^F isotope, most commonly used in positron emission tomography (PET), is produced in a cyclotron through bombarding the ^18^O-enriched water in the target casing with a beam of accelerated protons. Many factors are taken into account when designing the target body.

During the production of radionuclides, the irradiated target material in cyclotrons is most often in a liquid or gas form. During the bombardment with protons in these centers, rapid heating occurs, causing an increase in their volume and, in the case of liquid, even boiling [1,2,3,4]. In addition, the protons from the beam partially lose their energy on their way in the target’s structural elements, causing their intense heating. In practice, the working chamber of the target is a hole drilled in a cylinder made of metal. This metal must be relatively easy to machine but of a high strength and characterized by good thermal conductivity. The working chamber from the side of the beam entrance has a window made of a durable foil pressed against the working space of the target with an increased pressure, necessary to compensate for the effects of rapid heating of the irradiated medium. Increasing the strength of the target window by increasing the thickness of the foil would cause too much loss in the proton beam that passes through it. Therefore, this problem was solved by using an additional mesh supporting the thin foil. The most commonly used are Havar alloy foils with a high cobalt content supported by a mesh made of copper or aluminum [1,2,3]. Target bodies are usually made of silver, tantalum, titanium, or niobium [5,6,7,8].

The cooling of the target material and target body components is one of the main considerations when designing the target body. The target body made of silver, due to its high thermal conductivity of 415 W/(m·K), is characterized by a high heat exchange efficiency, whereas the thermal conductivity of tantalum is 57 W/(m·K), and that of titanium is only 21.9 W/(m·K). The analyzed cyclotron originally used a target body made of silver, which was then replaced with a tantalum body. The reduced heat transfer rate of tantalum was compensated for by the increased cooling of the rear face of the target body using an additional radiator. In addition, the irradiation of the water enriched in the silver body led to the formation of colloids, which resulted in a decrease in the efficiency of radionuclide production. In this case, the entire item had to be removed from the accelerator and cleaned to have its functionality restored. Making the target body composed of tantalum extended the life of the element and reduced the number of impurities in the liquid target material.

This work discusses the influence of the used target body on radionuclide contaminants in the irradiated material. Such impurities are most often formed when the beam passes through the target window, but they can also arise when protons with sufficient energy hit the walls of the target working chamber. Earlier studies analyzed the formation of such impurities in the target with a housing made of silver [9]; there is a discussion in the literature regarding isotopes activated in titanium and resulting from irradiation of the beam of protons accelerated in the cyclotron [10,11,12]. The activation processes resulting in the production of radionuclides also occur in the elements of linear medical accelerators in nuclear reactions caused by photons accelerated to high energies and secondary neutrons [13,14,15]. In the following paper, theoretically possible nuclear reactions with tantalum will be discussed, which result in the formation of stable and radioactive isotopes that are impurities during the production of isotopes for positron emission tomography. The following reactions were analyzed: (n,p), (p,2n), (p,a), (p,d), (n,p), and (n,g). Theoretical predictions will be compared with the results of gamma spectrometry measurements of the set of columns for the purification of fluorodeoxyglucose [^18^F]FDG.

## 2. Results

The results of the nuclear reactions (n,p), (p,2n), (p,a), (p,d), (n,p), and (n,g) with the isotopes from elements used in both target bodies were theoretically predicted. The tables below show the possible nuclear reactions taking place in target bodies together with the maximum value of the cross-sections of these reactions with the energy values. The analyzed energy range spans from the reaction threshold energy to the maximum proton energy of 11 MeV (Table 1 and Table 2). Natural silver contains two stable isotopes: ^107^Ag and ^109^Ag. The first isotope is 51.839%, and the second is 48.161%. Natural tantalum is 99.988% stable isotope ^181^Ta.

The predicted radioactive isotopes that can be formed in the natural silver target casing are ^107^Pd, ^109^Pd, ^106^Ag, ^108^Ag, ^109^Cd, and ^107^Cd (Table 1). The presence of the cadmium isotope ^109^Cd was confirmed in a previous study [9]. In the case of a tantalum target casing, possible radioactive impurities are isotopes ^181^W, ^180^W, and ^181^Hf (Table 2). The tests performed with gamma spectrometry confirmed the presence of theoretically predicted cadmium isotopes in the target made of silver in the samples from the production of FDG. Due to a very short half-life of the theoretically predicted silver isotopes, their presence could not be confirmed during measurements performed several dozen hours after the end of the irradiation. The presence of the theoretically predicted isotopes of palladium (^107^Pd, ^109^Pd) has also not been confirmed. These are isotopes decaying with the emission of the β-electron, characterized by single emission lines of gamma radiation, and their reaction cross-section is an order of magnitude lower than that of the confirmed cadmium isotopes.

**Figure 1:**
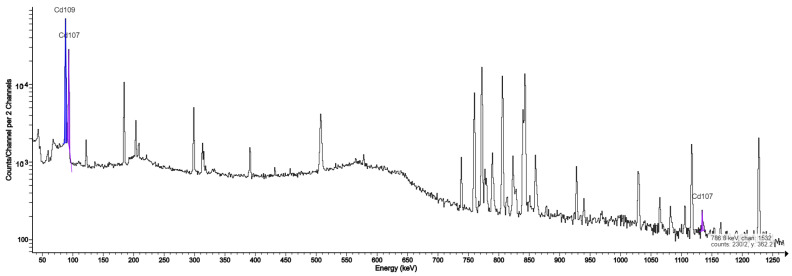
Gamma ray spectrum of the QMA column with visible gamma peaks characteristic of Cd-107, Cd-109 isotopes from FDG production using a silver target.

**Figure 2:**
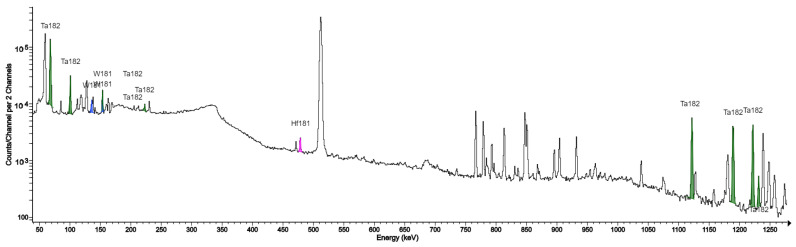
Gamma ray spectrum of the QMA column with visible gamma peaks characteristic of the isotopes W-181, W-180, and Hf-181 from FDG production using a tantalum target.

In our study, the target window was made of Havar alloy foil, and many radioisotope impurities originating from nuclear reactions between protons from the beam and secondary neutrons and atoms of elements constituting the Havar alloy were identified in the tested samples (Table 3). These are ^55,56,57^Co, ^95,95m,96^Tc, ^52^Mn, and ^182,182m,183,184,186^Re. Characteristic photon peaks for these radionuclides were confirmed in the spectrometric spectra of the set of columns used in the production of the target housings made of both silver and tantalum (Figure 1 and Figure 2). The presence of remium isotopes ^182^Re, ^182m^Re, ^183^Re, ^184^Re, ^184m^Re, and ^186^Re makes identification of ^182^Ta difficult because most of the peaks are characteristic of tantalum coincide with those of gamma radiation characteristic of at least one isotope of remium.

## 3. Materials and Methods

**Figure 3:**
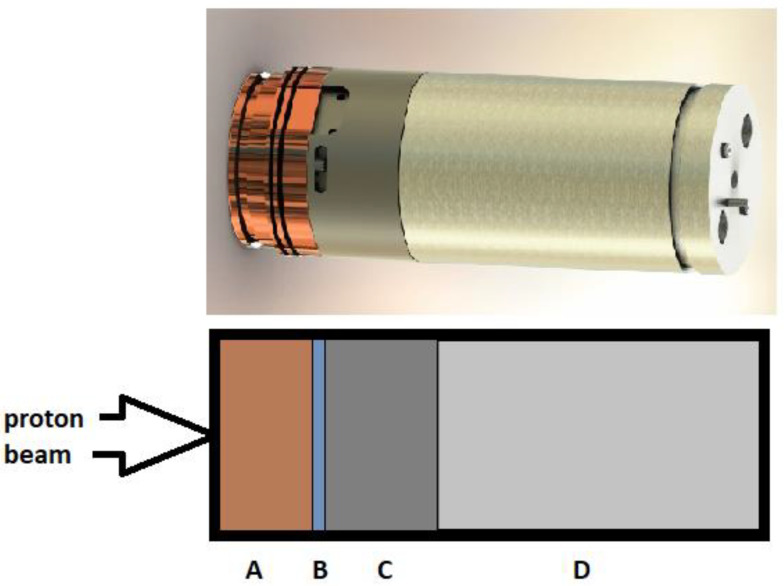
The target for the production of the F-18 (RDS 111 & HP ECLIPSE Target, Simens, Warsaw, Poland) and the scheme of its construction. A—Copper Hex Grid, B—target window (Havar foil), C—Target Body (silver or tantalum), D—Target Support Cylinder Set with radiator and water cooling.

The cyclotron used in the work is located in the Provincial Multi-Specialty Center of Oncology and Traumatology in Łódź [3,16,17]. The Eclipse™ RD cyclotron (Siemens, Warsow, Poland) used is a bare-shielded cyclotron that accelerates proton energy to 11 MeV and produces ^18^F and ^11^C positron-emitting radioisotopes. The ^18^F isotope is formed during the bombardment of ^18^O-enriched water in the ^18^O(p,n)^18^F reaction, and the target casing was initially made of silver and then replaced with another made of tantalum during the upgrade of the cyclotron [18]. Tantalum target for the production of F-18 was placed in a visible, empty working chamber and filled with oxygen-18-enriched water and irradiated during cyclotron operation (Figure 3).

Typical irradiation parameters include:

- Enrichment of ^18^O, typically >95%.
- Chemically pure enriched water ^18^O, above 99.99%.
- Target volume of 2.5 mL.
- A beam of protons with an energy of 11 MeV.
- Beam current of 60 μA.
- Irradiation time of 90 min.

An 11 MeV proton beam accelerated in a cyclotron passes through a 50 μm thick Havar window almost completely (99.999%), losing only 3.3 keV of its energy [19]. During the collisions, some of the protons from the beam change direction and hit the target’s body, causing nuclear side reactions that produce the radionuclide contaminants analyzed in this work. Protons mainly lose their energy in enriched water. However, this energy is sufficient to produce ^18^F in the ^18^O(p, n)^18^F reaction, because the cross-section of this reaction is maximal at 5–6 MeV.

In theoretical considerations, the open database system of the International Atomic Energy Agency (IAEA) TENDL-2019 (TALYS Evaluated Nuclear Data Library version 2019) was used to predict possible nuclear reactions, and the Nuclear Data Section database was used to determine the stability and decay methods of the resulting isotopes (https://www-nds.iaea.org (accessed on 7 February 2023)). Kernel model is a computer code applied for predicting and analyzing nuclear reactions. Code simulates reactions involving neutrons, gamma rays, protons, deuterons, tritons, helions, and alpha particles in the energy range from 1 keV to 200 MeV [20,21,22,23,24,25].

The radionuclide identity can be confirmed by obtaining a gamma spectrum or by measuring the half-life of the product. Therefore, for the identification and quantification of radioisotopes, a high-resolution, low-background gamma spectrometry system was used, equipped with an HPGe detector with a relative efficiency of up to 30%. The analysis was carried out using a dedicated Genie 2000 software and an ISOCS/Lab SOCS supporting software.

Samples from nine different days of routine fluorodeoxyglucose production were tested using a tantalum target housing with a Havar foil window. The tested samples were elements of the filtering system applied for the synthesis of [^18^F]FDG: a separation column used to capture fluorine ions from the water after irradiation—QMA (Quaternary ammonium anion exchange) and a set of filtration columns used to purify the finished FDG. The filter set consisted of five columns used in the on-chip set: IC-H (cation exchange resin), two IC-HCO3 (anion exchange resin), AL-N, and C18. Each of the column sets was used in only one production run. They were subjected to a spectrometric analysis 30–48 h after the end of the synthesis. The results were compared with the results of analogous five sets obtained in the production of FDG with the use of silver-body targets.

## 4. Discussion

In this work, a gamma radiation spectrometer was used for a quantitative and qualitative analysis of the elements of the FDG synthesis modules, such as a separation column and a set of filtration columns. Similar analyses were carried out for the target body made of silver with windows made of Havar foil, and the results of these analyses were described in the works of Ferguson (2011) [13], Bowden (2009) [14], and Marengo (2008) [20]. Mochizuki (2006) [11] also analyzed the elements of the synthesis modules, but for the target casing made of titanium, similarly to the team of Guarino et al. (2007) [12]. Meanwhile, Köhler (2013) [15] analyzed samples in the form of water enriched in ^18^O after irradiation in the production of FDG in a target with a window from niobium. Researchers using silver target casings identified the isotopes ^109^Cd, ^105^Ag, ^106m^Ag, and ^110m^Ag as radionuclide impurities, the source of which were reactions occurring in the immediate vicinity of the target (Table 4).

Among the isotopes indicated in this work, the presence of ^109^Cd and ^107^Cd detected in our previous studies was confirmed [3]. Two isotopes, ^46^Sc and ^48^V, were identified during the period of use of the titanium case. The sources of these radionuclide impurities are the reactions ^46^Ti(n,p)^46^Sc, ^48^Ti(p,n)^48^V, and ^48^Ti(p,2n)^48^V. Kambali et al. (2020) [10], in their work, predicted the possibility of generating additional 10 radioisotopes when using a titanium target casing: ^43,44,47Sc,45,46,47,49,50^V and ^45,51^Ti. Köhler, using a niobium target window, additionally identified isotopes ^89^Zr, ^92m^Nb, and ^93m^Mo. In this work, we are the first to confirm the presence of the indicated isotope impurities in practice during the production of FDG in the tantalum target casing as a source of radionuclide impurities and to quantitatively confirm their contribution. We had a unique opportunity to compare the effect of changing the target on the formation of impurities in practice, when after seven years of use, the silver target was replaced with an identical tantalum one, which has already been used for two years. Spectrometric analysis identified three new radionuclide impurities: ^181^W, ^181^Hf, and ^182^Ta. In the previous target, the emerging cadmium isotopes were the dominant impurity, which is very clearly visible in the presented picture (Figure 2). In the current system, the most dominant gamma-ray peak comes from ^182^Ta, while ^181^W and ^181^Hf are difficult to identify. The nuclear reaction cross-sections for the production of these two isotopes are incomparable to the cross-sections for the production of ^182^Ta.

The main source of radionuclide impurities arising during the production of ^18^F is the window closing the target working chamber. The type of formed radionuclides depends on the used material; the most common are Havar foils, but also niobium and titanium are used. The energy of the proton beam also affects the number of pollutants formed.

## 5. Conclusions

The sources of radiation in the region of cyclotron are the basic nuclear reactions (p,n) and (d,n). Examples of these reactions are ^18^O(p,n)^18^F, ^15^N(p,n)^15^O, ^12^C(d,n)^13^N, and ^14^N(d,n)^15^O. Neutron radiation is also observed during the production of the ^11^C isotope in the ^14^N(p, α)^11^C reaction in which no neutrons are produced. This is due to the fact that, in addition to the basic reactions, neutrons can be generated by other secondary reactions caused by the interaction with the elements of the cyclotron directly in the path of the beam of accelerated charged particles.

Among the analyzed metals, tantalum is the optimal material in terms of the production of radionuclide impurities during the production of ^18^F by the irradiation of ^18^O-enriched water with protons in accelerated cyclotrons. Only three radionuclides with half-lives of fewer than 120 days were identified in the tested samples. In the case of silver body target impurity, the half-life of the identified ^109^Cd is 462 days. Therefore, a new cyclotron body target based on tantalum has been evaluated, as it promises a new material potentially classified as intermediate or low-level radioactive waste in future.

## Figures and Tables

**Table 1:** Theoretical analysis of nuclear reactions with silver isotopes ^107^Ag and ^109^Ag.

Nuclear Reaction	Threshold Energy [MeV]	Maximum Cross-Section [mbarn]/Energy [MeV]	Decay Mode	Half-Life
^107^Ag(p,n)^107^Cd	2.219	519.354/11	EC, β+	6.5 h
^107^Ag(p,2n)^106^Cd	10.22	95.313/11	Stable	-
^107^Ag(p,a)^104^Pa	0	1.792/11	Stable	-
^107^Ag(p,d)^106^Ag	7.38	56,952/11	EC, β+	23.96 min
^107^Ag(n,g)^108^Ag	0	852/thermal neutrons	EC, β+	2.382 min
^107^Ag(n,p)^107^Pd	0	24.43/11	β−	6.5 × 10^6^ y
^109^Ag(p,n)^109^Cd	1.0071	394/9	EC, β+	462 d
^109^Ag(p,2n)^108^Cd	8.398	428/11	Stable	-
^109^Ag(p,a)^106^Pa	0	394/11	Stable	-
^109^Ag(p,d)^108^Ag	7.024	0.001/11	EC, β+	2.38 min
^109^Ag(n,g)^110^Ag	0	1,784,480/thermal neutrons	Stable	-
^109^Ag(n,p)^109^Pd	0.334	12.61/11	β−	13.59 h

**Table 2:** Theoretical analysis of nuclear reactions with tantalum isotope ^181^Ta.

Nuclear Reaction	Threshold Energy	Maximum Cross-Section [mbarn]/Energy [MeV]	Decay Mode	Half-Life
^181^Ta(p,n)^181^W	0.976	64.994/9	EC. β+	121.2 d
^181^Ta (p,2n)^180^W	7.699	398.1/11	Stable	-
^181^Ta (p,a)^178^Hf	8.86	0.0352/11	Stable	-
^181^Ta (p,d)^180^Ta	5.38	0.000009/11	Stable	-
^181^Ta (n,g)^182^Ta	0	1,784,480/thermal neutrons	EC. β+	114.74 d
^181^Ta (n,p)^181^Hf	0	0.493/thermal neutrons	β−	42.39 d

**Table 3:** Isotopes identified in the spectra with their characteristic peaks.

Isotope	Half-Life	Gamma Radiation/Intensity
^52^Mn	5.591 d	1434.09 (100%), 935.54 (94.5%), 744.23 (90%)
^55^Co	17.53 h	931.3 (75%), 477.2 (20.2%), 1408.5 (16.9%)
^56^Co	77.23 d	846.77 (99.9%), 1238.29 (66.46%), 2598.50 (16.97%), 1771.35 (15.41%), 1037.84 (14.05%)
^57^Co	271.74 d	122.06 (85.60%), 136.47 (10.68%)
^95^Tc	20 h	765.79 (93.82%)
^95m^Tc	61 d	204.117 (63.20%), 582.08 (29.96%), 835.149 (26.63%)
^96^Tc	4.28 d	778.224 (99.76%), 849.93 (98%), 812.58 (82%), 1126.85 (15.2%)
^107^Cd	6.5 h	93.12 (4.8%), 828.93 (0.17%)
^109^Cd	462.6 d	88.03 (3.61%)
^181^W	121.2 d	152.32 (0.08%), 136.28 (0.03%)
^181^Hf	43.39	482.18 (80.5%), 133.02 (43.3%), 345.92 (15.12%)
^182^Ta	114.43 d	67.749 (42.9%), 1121.3 (35.24%), 1221.4 (27.23 %), 1189.04 (16.49%)
^182^Re	64 h	229.32 (25.8%), 67.85 (22.2%), 1121.3 (22.1%), 1221.1 (17.5%), 100.1 (16.5%), 1231.01 (14.9%), 169.15 (11.4 %)
^182m^Re	14.14 h	67.75 (38.3%), 1121.4 (32%), 1221.5 (25%), 1189.2 (15.1%), 100.2 (14.4%)
^183^Re	70 d	162.33 (25.1%), 46.48 (8.0%)
^184^Re	38 d	903.28 (38.1%), 792.06 (37.7%), 111.21 (17.2%), 894.76 (15.7%)
^184m^Re	169 d	104.73 (13.4%)
^186^Re	3.718 d	137.15 (9.47%)

**Table 4:** List of radioactive impurities identified in target bodies by various research groups.

Autor	Point of Analysis	Target Foil/Target Body	Identified Importunities from Foil	Identified Importunities from Target Body
Ferguson [13]	Synthesis cassettes	Havar/silver	^51^Cr, ^52,54^Mn, ^56,57,58^Co, ^95m,96^Tc, ^182,183^Re	^109^Cd
Bowden [14]	Silver target body “in situ” in cyclotron, synthesis cassettes, sample of irradiated [^18^O]H_2_O, before and passing through the QMA column	Havar/silver	^51^Cr, ^52^Mn, ^56,57,58^Co, ^95m,96^Tc, ^183,184^Re	^110m^Ag ^109^Cd
Mochizuki [11]	Target foil, separation and filtration column	Havar/titan	^48^V, ^51^Cr, ^52,54^Mn, ^56,57,58,60^Co, ^95m,96^Tc, ^183,184^Re	^46^Sc, ^48^V
Guarino [12]	Target window foil (havar), titanium vacuum window	Havar and titanium/-	H: ^51^Cr, ^52,54^Mn, ^56,57,58^Co, Ti: ^46^Sc, ^48^V	Not analyzed
Köhler [15]	Sample of irradiated [^18^O]H_2_O	Niob/-	^48^V, ^51^Cr, ^52,54^Mn, ^55,56,57,58^Co, ^57^Ni, ^89^Zr, ^92m^Nb, ^93m^Mo, ^95,96^Tc,	Not analyzed
Marengo [20]	Synthesis cassettes, sample of collected [^18^O]H_2_O, purification filters	Havar/silver	^48^V, ^51^Cr, ^52,54,56^Mn, ^55,56,57,58,60^Co, ^57^Ni, ^95,95m,96,98^Tc, ^182,182m,183,184,186^Re	^105^Ag, ^106m^Ag, ^109^Cd
Długosz-Lisiecka [9]	Synthesis cassettes, QMA separation filter, purification filters	Havar/silver	Not analyzed	^107^Cd, ^109^Cd
in this work	QMA separation filter, purification filters	Havar/silver Havar/tantal	^52^Mn, ^55,56,57^Co, ^95,95m,96^Tc, ^182,182m,183,184,184m,186^Re	Ag: ^107^Cd, ^109^Cd Ta: ^181^W, ^181^Hf, ^182^Ta

## Data Availability

The data presented in this study are available on request from the corresponding author.
